# Earlier Menarche in Greek Girls Born by Caesarean Section: A Case–Control Study

**DOI:** 10.3390/jcm13123452

**Published:** 2024-06-13

**Authors:** Vasiliki Rengina Tsinopoulou, Eleni P. Kotanidou, Nikolaos Athanasiadis, Flora Bacopoulou, Charikleia Stefanaki, Liana Fidani, Assimina Galli-Tsinopoulou, Athanasios Christoforidis

**Affiliations:** 1 2nd Department of Pediatrics, School of Medicine, Faculty of Health Sciences, Aristotle University of Thessaloniki, University General Hospital AHEPA, 54636 Thessaloniki, Greece; 2 1st Department of Pediatrics, School of Medicine, Faculty of Health Sciences, Aristotle University of Thessaloniki, Ippokratio General Hospital, 54636 Thessaloniki, Greece; 3 3rd Department of Obstetrics and Gynecology, School of Medicine, Faculty of Health Sciences, Aristotle University of Thessaloniki, Ippokratio General Hospital, 54636 Thessaloniki, Greece; 4 Center for Adolescent Medicine and UNESCO Chair in Adolescent Health Care, First Department of Pediatrics, Medical School, National and Kapodistrian University of Athens, Aghia Sophia Children’s Hospital, 11527 Athens, Greece; 5 Laboratory of Genetics, School of Medicine, Faculty of Health Sciences, Aristotle University of Thessaloniki, 54636 Thessaloniki, Greece

**Keywords:** puberty, early menarche, menarcheal age, menarche, Greece, adolescents, pubertal timing, caesarean section

## Abstract

**Objectives:** The purpose of this study was to report on the menarcheal age in girls of Greek origin and assess its potential associations with their demographic and perinatal data, as well as their maternal menarcheal age. **Methods:** In this case–control study, adolescent girls were recruited between September 2021 and September 2022 from two Pediatric Endocrinology Units, Aristotle University of Thessaloniki, Greece. Eligible participants included Greek girls up to the age of 18 years, with menarche and the absence of chronic disease or chronic medication use. Participants were divided into two groups, the early menarche group and the control group (menarche before or after 11 years of age, respectively). Data included participants’ maternal menarcheal age, their chronological age, place of residence, anthropometric data (at recruitment) and perinatal data (birth order, gestational age, type of delivery, birth weight/length). **Results:** A total of 100 girls aged 7–17 years (mean age ± SD 12.51 ± 2.59 years) were included in this study. The mean ± SD menarcheal age of the total sample was 11.47 ± 1.55 years (median 11.20 years; range 7.50–16.25 years); 43% had early menarche (median menarcheal age 10.50 years; range 7.50–10.91 years), and 57% had menarche after age 11 (median menarcheal age 12.08 years; range 11.00–16.25 years). The caesarean section rate was significantly (*p* < 0.001) higher in girls with early menarche (83.7%) than controls, whereas other variables did not differ significantly between groups. **Conclusions:** This Greek sample demonstrated a relatively young age at menarche with a significant proportion of girls with early menarche; in the latter group, the rate of caesarian sections was significantly higher than controls.

## 1. Introduction

The milestones of puberty in girls are the time of onset, the growth spurt and age of menarche. The onset of puberty is characterized by the development of the breast bud, while the growth spurt represents the period of the highest growth velocity as a result of the synergistic action of sex steroids and growth hormone. Menarche is defined as the presence of the first vaginal bleeding and is usually signaling the end of puberty in girls [1].

Early menarche is considered that occurring before the chronological age of 11 years, while late menarche is considered that occurring after the chronological age of 15 years [2]. The deviation of the age at menarche either early or late is related to a series of effects on the health of women [3,4,5]. According to an umbrella review, young age at menarche (<12 years), when compared with menarche after the age of 12, was associated with complex cardiovascular morbidity in later life, especially ischemic heart disease but not stroke mortality [3]. Moreover, according to another umbrella review, early menarche is a possible marker of metabolic disorders (metabolic syndrome, impaired glucose metabolism, insulin resistance, type 2 diabetes mellitus, gestational diabetes, hypertension), cancers (breast, endometrial, ovarian) and an increased death risk [6]. 

In the Greek population, the average age of menarche is 12.29 years [7], with no particular changes over a decade (average age over a decade, 12.27 years) [8]. The age at which menarche occurs depends on the interaction of various factors, both genetic and environmental, as well as largely unknown factors [9,10,11,12]. The age at which the initiation of puberty occurs is determined by 50–80% genetic factors [13], while prenatal life and perinatal environment, along with factors impacting these periods of life, affect directly or indirectly the age at menarche. The most important factors studied to date are birth weight (especially small for gestational age), month of birth, method of delivery (vaginal or caesarean section), gestational age, birth order (first birth, second birth), size of the family, environment (urban or not) and maternal menarcheal age [14,15,16,17,18,19]. Depending on the definitions used, up to 10% of all live-born neonates are small for gestational age (SGA), and while most of these children demonstrate catch-up growth by 2 years of age, one in ten does not [20]. Interestingly, SGA birth carries an increased risk of morbidity. Furthermore, low birth weight can persevere in two consecutive generations; a low-birth weight mother has 2.8 times the risk of giving birth to a low-birth weight baby [21]. Numerous epidemiological studies in animals and humans have reported that early life events lead to an increase in non-communicable chronic diseases in adulthood, such as obesity, type 2 diabetes mellitus and cardiovascular disease [22]. These children should be monitored for weight gain, signs of adrenarche and puberty and metabolic and reproductive abnormalities [23]. On the other hand, there is a lack of agreement on the association between the mode of delivery (caesarian section versus vaginal delivery) and the appearance of early puberty and consecutively early menarche. 

The purpose of the present study was to report on the menarcheal age in girls of Greek origin and assess its potential associations with their demographic and perinatal data, as well as their maternal menarcheal age.

## 2. Materials and Methods

In this case–control study, adolescent girls were recruited from September 2021 to September 2022 from the Pediatric Endocrinology and Metabolism Unit of the Second Department of Pediatrics of the Aristotle University of Thessaloniki (AUTH) at the AHEPA University Hospital and the Pediatric Endocrinology Unit of First Department of Pediatrics of AUTH at the Ippokratio General Hospital of Thessaloniki, in Greece. During recruitment, girls and their parents or guardians (mother and/or father) were fully informed about the purpose and methods of this study and gave their written consent to participate. Adolescents up to 18 years who had spontaneous menarche regardless of the age of commencement were eligible to enter the study. Girls with anthropometric data (weight, height and body mass index) above the 95th percentile for their age and those with chronic disease or under chronic medication were excluded. Girls with a history of any condition that could have affected the progression of puberty (i.e., hypothyroidism, growth hormone deficiency, etc.) and those who had received treatment for pubertal induction or delay were also excluded. 

Data included participants’ chronological age, place of residence (urban or rural) and anthropometric data at the time of recruitment. Perinatal history was retrieved from each participant’s health care records, and data such as birth order, gestational age, type of delivery (caesarean section or vaginal delivery), birth weight and birth length were also recorded. In relation to their birth weight, participants were classified as born small for gestational age (SGA) if their birth weight was below the 10th percentile, appropriate for gestational age (AGA) or large for gestational age (LGA) if their birth weight was above the 90th percentile [24,25]. Participant and maternal menarcheal age (reported from each participant’s mother) was categorized as early menarche (before the age of 11 years) or normal menarche (after the age of 11 years) [2]. 

The present study is part of a wider study of genes related to menstruation and has received approval from the Bioethics Committee of the School of Medicine, Faculty of Health Sciences of the AUTH (Protocol No. 3.297, 3/22.12.2020). 

This study was designed following the STROBE Statement—checklist for observational studies (https://www.strobe-statement.org, accessed on 9 September 2022).

### Statistical Analysis

The Statistical Package for Social Sciences, SPSS^®^, version 25 (IBM Statistical Package for Social Sciences for Windows, Version 25.0. Armonk, NY, USA, IBM Corp), was used for statistical analyses. Testing for the normality of study parameters was performed with the Kolmogorov–Smirnov and Shapiro–Wilk tests. To compare the frequencies of categorical variables, Fisher’s exact test or the chi-square test was used. Logistic regression was performed using the “Enter” method. The limit of statistical significance was set at 5% (*p* < 0.05).

## 3. Results

**Figure 1:**
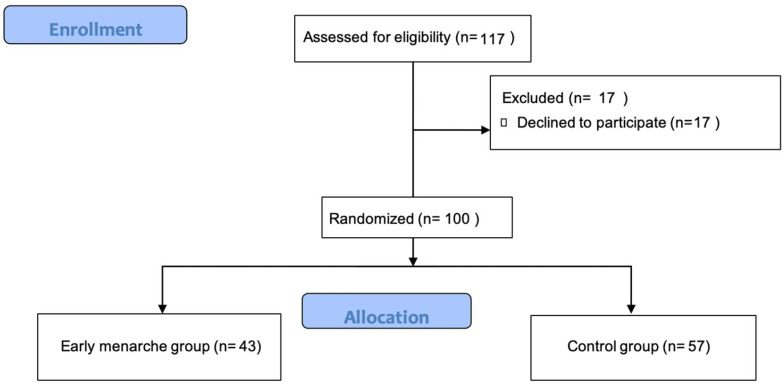
Flowchart of participants’ allocation.

During the study period, 117 girls were deemed eligible to participate in the study, from which 100 adolescent girls agreed to participate (Figure 1). 

Participants were 7 to 17 years old (mean age ± SD 12.51 ± 2.59 years), and 57% of them were residing in urban areas at enrollment. The mean (±SD) menarcheal age of the total sample was 11.47 (±1.55) years, and the median menarcheal age was 11.20 years, whereas menarcheal age ranged from 7.50 to 16.25 years. Regarding maternal menarcheal age, 76% of participants’ mothers reported menarche after the age of 11 years, while 24% reported early menarche before the age of 11 years. 

Participants’ characteristics at birth and at enrollment are presented in Table 1.

Participating girls were divided into two groups: the early menarche group, with menarcheal age before the chronological age of 11 years, and the control group with menarcheal age after 11 years. Of the total sample, 43% of the participants had early menarche with a median menarcheal age of 10.50 years (range 7.50 to 10.91 years), while 57% had menarche after the age of 11 years with a median menarcheal age of 12.08 years (range 11.00 to 16.25 years). 

A multiple logistic regression was conducted with the dependent variable as the early menarche group vs. the control group and independent variables as maternal menarcheal age, birth weight, birth length, birth order, method of delivery and area of residence. Analysis revealed the delivery method as the only statistically significant variable (*p* < 0.001), whereas the other variables did not differ significantly between the two study groups, as shown in Table 2. 

Comparing the frequency of early maternal menarcheal age, no difference was observed between the two groups (*p* = 0.241, OR: 0.55, 95% CI: 0.22–1.39). More precisely, among participants with early menarche, 30.2% had a mother who also had early menarche (*n* = 13/43), whereas 69.8% had a mother with menarche after the age of 11 years (*n* = 30/43). Among girls of the control group, 19.3% (*n* = 11/57) had a mother with early menarche, whereas 80.7% of girls had a mother with menarche after the age of 11 years (*n* = 46/57). 

Regarding participants’ birth weight, non-significant differences were found in the frequency distribution of SGA, LGA and AGA between the two study groups. More specifically, 16.3% of girls with early menarche were born SGA in contrast to girls with menarche over 11 years, where SGA birth was recorded in 22.8% (*p* = 0.460, OR: 1.52, 95% CI: 0.55–4.21). Similar findings were also obtained for those girls born LGA, who constituted 4.7% of the early menarche group compared to 8.8% of those in the control group (*p* = 0.695, OR: 1.97, 95% CI: 0.36–10.68). Girls born AGA, on the contrary (*p* = 0.263, OR: 0.57, 95% CI: 0.23–1.44), constituted a higher percentage (68.4%) of the control group compared to the early menarche group (79.1%). 

The method of delivery presented a statistically significant difference between the two groups, with the rate of caesarean section in girls with early menarche being significantly higher (83.7%) than in controls (21.1%) (*p* < 0.001, OR: 0.05, 95% CI: 0.02–0.15). Conversely, vaginal delivery rates were higher in the control group (78.9%) than in the early menarche group (16.3%). 

Finally, the area of residence, birth length and birth order did not differ significantly between the two study groups (Table 2).

## 4. Discussion

The present study aimed to report the up-to-date menarcheal timing of a female sample of Greek origin and its correlation with demographic and perinatal data, as well as maternal menarcheal age.

The median menarcheal age of our study sample was 11.20 years, with more than two out of five girls reporting early menarche, thus demonstrating a decreasing trend in the age of menarche when compared to previous Greek studies [1,7,8,26] and a relatively recent 5-year study in Athens [27].

Most mothers reported a normal age of menarche (older than 11 years), while there was no statistically significant correlation between the menarcheal age of participants and that of their mothers, a finding that is not supported by the literature and could probably be attributed to the small number of study participants. It should be noted that maternal age at menarche has been positively associated with the timing of puberty in both boys and girls, but the correlations are particularly strong in girls, especially with their menarcheal age [28]. 

Despite evidence relating early puberty/menarche with birth order [18,29], we found no such association in our Greek study sample.

There is also evidence about the earlier onset of menarche in girls from urban areas than girls living in rural areas, an observation that is explained by the different exposures to environmental factors that may play a role in accelerating ovulation and the levels of stress [11,28]. Furthermore, according to a recent literature review, menarche is dependent on the level and duration of exposure to chemicals, as well as on intrauterine or antenatal exposure [30]. A similar difference was not found in the present study despite the fact that most participants lived in an urban environment. 

The present study failed to demonstrate any association of menarcheal age with the birth weight status (SGA, LGA or AGA). The results are not in line with studies reporting early thelarche, early adrenarche and early menarche in girls born SGA vs. AGA [1]. However, to date, the causal relationship between SGA birth status and early menarche has not been clearly demonstrated, while the most likely explanation seems to be the role of increased levels of leptin, the classic adipose tissue hormone. Specifically, the catch-up growth of SGA neonates in the first years of life, which is accompanied by a rapid increase in body weight, is characterized by an increase in adipose tissue and leptin levels, which directly act on the neurons that secrete the gonadotropin-releasing hormone (GnRH) and lead to their early activation and consequently to early onset of puberty [31,32]. In addition, it is reported that leptin receptors, apart from pituitary gonadotropic cells, are also found in kisspeptin-producing cells [33], which in turn play an important role in the onset of puberty, as well as in ovarian follicular cells and Leydig cells [34].

Furthermore, research data show a delay in the puberty and menarche of girls born LGA compared to girls born AGA, and this is really an unanswered question to date [35]. On the contrary, boys born either SGA or LGA present earlier puberty than boys born AGA [35]. The present study failed to demonstrate a similar observation in the small number of girls born LGA.

According to the current literature, early menstruation is associated with a series of effects and complications for the health of adult women [3]. Early and prolonged exposure to estrogen is associated with an increased risk of neoplasms (breast, ovary, endometrium) [36,37], with even one-year delay in the occurrence of menarche to be considered as a protective factor [38]. There are also studies associating early menarche with a risk for developing polycystic ovary syndrome, metabolic syndrome, hyperinsulinemia and even cardiovascular events [36,39,40]. 

Furthermore, girls with early menarche may experience phycological frustration and disharmony, a feeling that occurs due to the divergence between psychic immaturity and physical maturity. Indeed, studies of adolescents with early menarche have shown higher percentages of depression and anti-social behavior in adulthood, when compared to adolescents with normal menarche [41]. Behavioral disorders, such as eating disorders, panic attacks and depression, tend to occur more frequently in girls with precocious puberty and/or menarche [37]. Moreover, girls with early menarche appear to be sexually active earlier than those with normal menarche [4] and at increased risk for adolescent dating abuse [42].

On the other hand, delay in the onset of menarche is associated with reduced bone density and inability to reach peak bone mass and, by extrapolation, with osteoporosis and an increased risk of fractures at all ages [39,43]. As with early menarche, delayed menarche can also increase the cardiometabolic risk of women, since delayed exposure to sex hormones can affect lipid metabolism, body mass index and cardiac function; nevertheless, early age at menarche (≤10 and 11 years) seems to have the greatest burden of major adverse cardiovascular events (40% and 27%, respectively) with the earliest age at menarche associated with the worst outcomes [44]. 

It is worth noting that some researchers suggest for girls born SGA, even with normal menstruation, close monitoring for the progress of their growth and their metabolic profile, as they often present disorders of growth and glucose metabolism (insulin resistance) as well as polycystic ovary syndrome [31,35,45]. 

An interesting finding that emerges from the present study is the significantly increased rate of caesarian sections in the group of girls with early menarche. Few studies have investigated the relation between the mode of delivery and the age of menarche. Huang et al. [19] found no significant association of caesarian section with menarcheal age. The authors, however, based their hypothesis on the transient inhibition of cortisol action, due to a reduction in perinatal stress during caesarean section and the consequent inhibition of the repressive action of stress on the hypothalamic–pituitary–gonadal axis [46]. Moreover, Aris et al. [47] studied the onset of puberty in a large number of boys and girls and found a trend towards an earlier onset of puberty in boys born by caesarian section compared to those born by vaginal delivery; however, no signs of earlier puberty were noted in the group of girls born by caesarian section [47]. 

A potential effect of caesarean section through epigenetics on several systems that justify a significant diversity of the gut microbiome that promotes obesity and early puberty [48] may explain our findings.

The above-mentioned hypothesis is well described in the literature in order to explain the potential role of the mode of delivery in child outcome [49]. Indeed, the literature mentions three distinct possible biological mechanisms that could explain the impact of the mode of delivery in infants and children. One hypothesis is based on the different immunological development of newborns born by caesarian section, due to inadequate exposure to maternal microbiota. This reduced exposure explains in a way the increased risk of immunological diseases in newborns born by caesarian section, such as bronchial asthma, ulcerative colitis and diabetes mellitus type 1, as well as the increased risk of metabolic disease and obesity [50]. Another hypothesis regarding the different health outcomes between children born by caesarian section and vaginal delivery is based on the great and nearly undiscovered, yet, area of interest, the so-called epigenetic impact on the genome of those children. Dahlen et al. [51] in their published medical hypothesis speculated that physiological labor has a protective effect in means of the epigenome on specific genes, mostly those responsible of programming immune responses, weight regulation and tumor suppression genes. The authors name physiological labor as a ‘eustressor’ capable of affecting the fetal genome in means of better efficiency and response to extrauterine life [51]. 

In the study of Black et al. [52], the health outcomes of 321,287 offsprings of primiparous women delivered either by caesarean section or vaginal delivery were followed-up. Their study showed an increased risk of asthma in offsprings after planned caesarean section compared to vaginal birth. On the other hand, a small but significant risk of diabetes mellitus type 1 was reported, even though the authors declared that this finding may contribute to a type I error. Last but not least, in coherence with the existing literature [53], the results of the study of Black et al. did not support an association between caesarean section and the risk of childhood cancer. 

Regarding the risk of childhood overweight and obesity and the association with the mode of delivery, rising evidence suggests a link of caesarean section with an increased risk of childhood obesity. A recent Canadian study by Bridgman et al. [54] showed that children born by cesarean section had increased odds of overweight by the age of 1 year, even though this rise did not persist by the age of 5 years. Nonetheless, a meta-analysis of twelve studies by Chiavarini et al. [55] revealed that children born by caesarean section are at an increased risk of overweight and/or obesity even beyond childhood to adulthood (risk 1.23, 95% CI, 1.09–1.39). 

Another interesting cohort study of Bräuner et al. [56] suggests that the exposure of a pregnant women to at least one stressful event could affect the age at menarche. In fact, exposure to just one stressful event is linked to menarche commencing 3.4 months earlier, while exposure to two or more stressful event increases the risk of an earlier commencement of menarche by 1.7 months. In their study, the authors suggest a possible mechanism of rising glucocorticoid levels, especially cortisol, during a stressful event passing through the placenta and the exposure of a growing fetus to those levels. Interestingly, the association between prenatal exposure to stress and age at menarche seems independent of the body mass index during childhood or adolescence [55]. 

The main limitation of the present study is the small sample; however, the findings lay ground for further study. Also, participants’ anthropometric data at menarche were not available, since most of them were recruited in this study at a later time. Furthermore, there were no data regarding participants’ weight gain during the early years of life.

In conclusion, the Greek sample studied demonstrated a relatively young age at menarche, whereas a significant proportion of girls had early menarche, and in this group of girls, the rate of caesarian sections was significantly higher than in control girls. Future studies of large population samples, exploring causal associations between the perinatal period and the timing of menarche, are warranted to confirm these observations. 

## Figures and Tables

**Table 1:** Participants’ characteristics (*n* = 100).

Characteristic	
Birth Timepoint	
Gestational age (weeks) [mean (SD)]	38.48 (1.55)
Birth weight (grams) [mean (SD)]	3115 (454.4)
Classification by birth weight	
*Small for gestational age (SGA) [n (%)]*	20 (20.0)
*Appropriate for gestational age (AGA) [n (%)]*	73 (73.0)
*Large for gestational age (LGA) [n (%)]*	7 (7.0)
Birth length (cm) [mean (SD)]	49.34 (2.82)
Delivery [*n* (%)]	
*Cesarean section*	48 (48.0)
*Vaginal delivery*	52 (52.0)
Birth order, *n* (%)	
*I*	63 (63.0)
*II*	25 (25.0)
*III*	11 (11.0)
*IV*	1 (1.0)
Study Stratification Timepoint	
Age (years) [mean (SD)]	12.51 (2.59)
Weight (kilos) [mean (SD)]	53.92 (18.67)
Height (cm) [mean (SD)]	152.14 (11.7)
Body mass index [mean (SD)]	22.76 (6.33)

SD, standard deviation.

**Table 2:** Differences in participants’ characteristics between two groups (early menarche group vs. control group).

*Characteristic* *(p Value) [OR (95% CI)]*	Early Menarche Group (n = 43)	Control Group (n = 57)
*Maternal menarche* *(p = 0.241) [0.55 (0.22–1.39)]*		
After 11 years	30 (69.8%)	46 (80.7%)
Early (before 11 years)	13 (30.2%)	11 (19.3%)
*Small for gestational age (SGA) (p = 0.460)* *[1.52 (0.55–4.21)]*		
Yes	7 (16.3%)	13 (22.8%)
No	36 (83.7%)	44 (77.2%)
*Appropriate for gestational age (AGA)* *(p = 0.263) [0.57 (0.23–1.44)]*		
Yes	34 (79.1%)	39 (68.4%)
No	9 (20.9%)	18 (31.6%)
*Large for gestational age (LGA)* *(p = 0.695) [1.97 (0.36–10.68)]*		
Yes	2 (4.7%)	5 (8.8%)
No	41 (95.3%)	52 (91.2%)
*Delivery method* *(p < 0.001) [0.05 (0.02–0.15)]*		
Cesarean section	36 (83.7%)	12 (21.1%)
Vaginal	7 (16.3%)	45 (78.9%)
*Area of residence* *(p = 0.221) [1.80 (0.80–4.07)]*		
Urban	28 (65.1%)	29 (50.9%)
Rural	15 (34.9%)	28 (49.1%)
*Birth order* *(p = 0.795)*		
I	24 (55.8%)	35 (61.4%)
II	11 (25.6%)	14 (24.6%)
Other	8 (18.6%)	8 (14.0%)

OR: Odds Ratio, 95% CI: 95% Confidence Interval.

## Data Availability

The datasets presented in this article are available from the corresponding author upon request.
